# From the One Health Perspective: Schistosomiasis Japonica and Flooding

**DOI:** 10.3390/pathogens10121538

**Published:** 2021-11-25

**Authors:** Su-Ying Guo, Lu Li, Li-Juan Zhang, Yin-Long Li, Shi-Zhu Li, Jing Xu

**Affiliations:** National Institute of Parasitic Diseases, Chinese Center for Disease Control and Prevention (Chinese Center for Tropical Diseases Research), NHC Key Laboratory of Parasite and Vector Biology, WHO Collaborating Centre for Tropical Diseases, National Center for International Research on Tropical Diseases, Shanghai 200025, China; guosy@nipd.chinacdc.cn (S.-Y.G.); Lilumyname@163.com (L.L.); zhanglj@nipd.chinacdc.cn (L.-J.Z.); liyl@nipd.chinacdc.cn (Y.-L.L.); Lisz@chinacdc.cn (S.-Z.L.)

**Keywords:** flooding, schistosomiasis, one health, environment

## Abstract

Schistosomiasis is a water-borne parasitic disease distributed worldwide, while schistosomiasis japonica localizes in the People’s Republic of China, the Philippines, and a few regions of Indonesia. Although significant achievements have been obtained in these endemic countries, great challenges still exist to reach the elimination of schistosomiasis japonica, as the occurrence of flooding can lead to several adverse consequences on the prevalence of schistosomiasis. This review summarizes the influence of flooding on the transmission of schistosomiasis japonica and interventions responding to the adverse impacts from the One Health perspective in human beings, animals, and the environment. For human and animals, behavioral changes and the damage of water conservancy and sanitary facilities will increase the intensity of water contact. For the environment, the density of *Oncomelania* snails significantly increases from the third year after flooding, and the snail habitats can be enlarged due to active and passive diffusion. With more water contact of human and other reservoir hosts, and larger snail habitats with higher density of living snails, the transmission risk of schistosomiasis increases under the influence of flooding. With the agenda set for global schistosomiasis elimination, interventions from the One Health perspective are put forward to respond to the impacts of increased flooding. For human beings, conducting health education to increase the consciousness of self-protection, preventive chemotherapy for high-risk populations, supply of safe water, early case finding, timely reporting, and treating cases will protect people from infection and prevent the outbreak of schistosomiasis. For animals, culling susceptible domestic animals, herding livestock in snail-free areas, treating livestock with infection or at high risk of infection, harmless treatment of animal feces to avoid water contamination, and monitoring the infection status of wild animals in flooding areas are important to cut off the transmission chain from the resources. For the environment, early warning of flooding, setting up warning signs and killing cercaria in risk areas during and post flooding, reconstructing damaged water conservancy facilities, developing hygiene and sanitary facilities, conducting snail surveys, using molluscicide, and predicting areas with high risk of schistosomiasis transmission after flooding all contribute to reducing the transmission risk of schistosomiasis. These strategies need the cooperation of the ministry of health, meteorological administration, water resources, agriculture, and forestry to achieve the goal of minimizing the impact of flooding on the transmission of schistosomiasis. In conclusion, flooding is one of the important factors affecting the transmission of schistosomiasis japonica. Multi-sectoral cooperation is needed to effectively prevent and control the adverse impacts of flooding on human beings, animals, and the environment.

## 1. Introduction

Schistosomiasis is a widespread water-borne parasitic disease at a global level, caused by six species of the parasite *Schistosoma*: *S. mansoni*, *S. japonicum*, *S. haematobium*, *S. mekongi*, *S. intercalatum* and *S. guineensis*. It affects more than 240 million people worldwide and more than 700 million people live in endemic areas [1]. The adult schistosome worm resides in the portal system of the liver, mesenteric arteries, the vessels of the bladder, and the genital tract of its definitive hosts [2]. Partial eggs retain in the host tissues to cause damage, whereas other eggs are discharged into the environment with feces or urine. The discharged eggs hatch and release free-swimming miracidia to penetrate specific snail hosts. The miracidia then develop in the snails, including two generations of sporocysts through asexual propagation, and shed abundant cercariae into fresh water. People become infected when they make contact with fresh water infested with cercariae. The cercariae penetrate the skin of the hosts and become schistosomulae, migrate through the circulation systems, and mature into adults in hosts’ specific organ and tissue [3,4].

Being a parasitic zoonosis, schistosomiasis japonica is prevalent in the Peoples’ Republic of China, the Philippines, and a few regions of Indonesia [1], which is transmitted through a wide spectrum of mammalian hosts but a strict intermediate host of *Oncomelania* spp. The life cycle reveals its dependence on the existence of the hosts and a decent environment. Thus, the prevention and control of schistosomiasis, which aims to disrupt its complex life cycle, should focus on all of the influencing factors in the transmission chain, including the hosts and the environment.

One Health is a global strategy combining the health of people, animals, and the environment and employs a holistic approach encouraging and expanding multidisciplinary collaborations, integrative research, capacity building, clinical practice, policy, and communication among many stakeholders [5,6]. This strategy is suitable for the prevention and control of schistosomiasis. The ecological distribution of schistosome during its growth and development depends on the geographic distribution of snails [7,8]. The distribution of the snails is related to several environmental factors, such as temperature, surface humidity, vegetation index, seasonality, and extreme events [9]. Flooding is an extreme event which has considerable impacts on the distribution of the snails and the transmission risks of schistosomiasis. In this review, we take a One Health perspective on flooding and its impacts on the transmission of schistosomiasis and summarize the One Health approaches to address the adverse impacts of flooding.

## 2. The Impact of Flooding on the Transmission of Schistosomiasis

Flooding is one of the major natural disasters that has brought tremendous losses to mankind in the last century by disrupting social growth and economic development [10,11,12]. The World Meteorological Organization reported that floods have caused 58,700 deaths and economic losses of nearly USD 115 billion over the past 50 years [13]. In recent decades, flooding occurred more frequently than before due to climate change and urbanization [12]. A study developed an integrated multivariate trend-frequency analysis (IMTFA) approach to assess climate extremes under global warming. A significant wetting and warming trend was found in Central Asia during the period of 1881–2018, and the warming trend significantly affected the intensities and frequencies of extreme precipitations in most regions of Central Asia [14]. In addition, several studies reported increasing trends in flood discharges in western Europe in the past five decades [15], including a 44% increase in the occurrence of extreme flood discharges [16]. Similar trends in the magnitude and frequency of flooding were also found in Brazil [17]. During 1903–2015, a significant fivefold increase in flood frequency in Amazonia is observable, from roughly one flood every 20 years during the first half of the 20th century to one flood every four years from the 2000s onward [18]. A review analyzed the floods in East China during 1984–2010 and reported a statistically significant increase of 77.4% in the flood cases per decade [19]. Although infrastructures have been strengthened in recent years to decrease the impacts of flooding, two catastrophic floods occurred along the Yangtze River basin in China in 2016 and 2020 [20,21]. The flood in 2020 resulted in 142 deaths/missing cases and a direct economic loss of around 116 billion RMB (~16.5 billion USD), though this is much better than the similarly destructive flood in 1998 due to the development construction of large-scale water conservancy projects on the Yangtze River’s mainstream and its tributaries [20].

Except deaths and economic losses, flooding can also increase the transmission risk of infectious diseases [22]. After flooding, traumatic injury, exposure to contaminated environment, poor hygiene, inadequate sanitation, and the lack of access to clean water would increase host vulnerability to various communicable diseases, such as bacterial diarrheal illnesses, cholera, hepatitis A virus infections, cryptosporidiosis, vector-borne diseases, and water-borne diseases [11,23,24,25,26,27,28]. Being a water-borne disease, the transmission of schistosomiasis japonica is also affected by flooding from the aspects of human beings, animal hosts, and environmental health [29].

### 2.1. The Impact of Flooding on Schistosomiasis Japonica in Human Beings

The occurrence of flooding will change the behavior of human beings and increase the intensity of water contact. Residents in rural areas could suffer from worse consequences because mostly they are nearer to the rivers than residents in the cities. Currently, most published studies of the flood-impact on water contact are carried out in China. Therefore, taking evidence from China for example, there are three reasons for the increase in water contact. First, flooding damages the embankments and people who previously live inside the embankments are directly exposed to the large amount of water that may contain schistosome cercariae. In the past 30 years, several catastrophic floods occurred along the Yangtze River basin in China. During the destructive flood in 1991, 14 endemic counties of schistosomiasis japonica in Hubei province were inundated, with more than 200 embankments collapsed and more than nine million of the population exposed to the contaminated waterbody [30]. Second, risk of water contact comes with various specific flood-related activities, such as flood rescue and self-relieved measures [31]. These activities could provide an extra chance of water contact and lead to an extra risk of infection, because people will not take these actions in usual years without flooding. During the flood in 1998 in China, over eight million people were exposed to the flood water [32], and nearly 150,000 soldiers and civilians participated in the flood fighting and rescue [33]. These soldiers and civilians were not supposed to be exposed to the fecal contaminated water in normal and dry years. Third, other human behaviors also change as a result of flooding and the frequency and duration of water contact alter. Evidence revealed that the major pattern of water contact during flooding was daily life contact in adult females, and swimming and paddling in adult males and children under 18 years old [34]. Certain individuals may attend some water-related recreational activities during flooding because the water body is enlarged by flooding. It should be noted that some amusement activities, such as swimming and fishing in the unknown wild open water, will increase infection risk, since flooding could even carry infected snails or cercariae to previous non-endemic areas of schistosomiasis. Thus, under the condition of higher intensity of water contact, the incidence risk of schistosomiasis will rise. Research reported that, compared with the control group, people in the case group of schistosomiasis had a higher frequency, longer accumulating time, and larger body contact surface area of the water contact [34].

In addition to the higher level of water contact, the dwelling environment and sanitation conditions of the local residents in the flood plains can be greatly influenced by flooding, which is also associated with increased infection risk. The damage to water and sanitation infrastructures during flooding may lead to the disruption of sewage disposal, poor standards of hygiene, poor nutrition, and negligible sanitation, which provide suitable conditions for the transmission of infectious diseases [11,35]. The lack of basic hygiene and sanitation and population displacement will also carry out the outbreak of water-borne and vector-borne diseases, such as schistosomiasis [36,37]. Inadequate feces management will lead to the discharge of feces containing schistosome eggs into the environment. Due to the lack of access to clean water, the victims of flooding are more likely to contact the contaminated water with cercariae.

More water contact leads to the increase in the prevalence of schistosomiasis. A study explored that the average number of acute cases in flood years was 2.8 folds higher than in other non-flood years [38]. The acute cases in Anhui [39] and Hubei [40] provinces increased by 61.73% and 43.90%, from 81 and 246 cases in 1997 to 131 and 354 cases in 1998, respectively, after the catastrophic flood in 1998. In addition, increases in the prevalence of schistosomiasis were also observed in Anhui, Hubei, Jiangxi, and Hunan provinces in 1999 after the 1998 flood [39,40,41,42,43,44,45]. Along with the development of the sanitation and health facilities and the improvement of the natural disaster emergency responses, there was no research reporting acute infection cases or cases with positive stool test results after the similar catastrophic flood in China in 2016, but an increasing trend was detected in the positive rate of indirect hemagglutination assay (IHA). The positive IHA rate of local residents in 26 national surveillance sites in Jiangxi province rose from 4.72% in 2016 to 5.58% in 2017 [46]. Similar positive association between rainfall and higher prevalence is also observed in the Visayas, the Philippines, whereas the relationship is negative in Mindanao with different topography [47]. This is a hint that the impact of flooding on the prevalence can be inverse when adjusting the topography factor. Future studies should explore the reasons of association between lower rainfall and higher prevalence in certain areas, which may provide critical evidence for policy makers targeting world-wide elimination of schistosomiasis. Besides, more evidence is needed to fill up the gap to identify the current status of threats of schistosomiasis transmission caused by flooding in the past 20 years.

### 2.2. The Impact of Flooding on Schistosomiasis Japonica in Animals

Similar to humans, the risk of schistosome infection in animals is on the rise with the occurrence of flooding. There are over 40 mammalian species serving as the reservoir hosts of *S. japonicum*, with bovines being the most important [48]. In flood plains, vegetation may change in terms of community structure, population size and phenology [49]. For domestic animals, they can be attracted by the vegetation on marshlands and riverbanks, because the grass is lush under the effect of flooding. Moreover, flood water can be contaminated by feces carrying eggs, and then the potentially contaminated flood water will invade grazing areas alongside the embankments due to the damage to barns. In the meantime, feces are also more likely to be excreted into flood water in grazing areas. These two factors will together lead to the increase in exposure risk for the animals. Consistent with the trend in human prevalence of schistosomiasis, the prevalence among the cattle in four counties in Hubei province increased about 1.68 folds in 1999 after the catastrophic flood in 1998 [40]. For wild animals, heavy rainfall encourages wild grass production which supports the growing of outdoor wild animal populations, such as rodents, and the flooding forces the animals from their burrows into closer areas with human beings due to the growing population and the losses of previous natural habitats [50]. The potentially infected wild animals will carry the worm to areas where schistosomiasis had been under control or even to previously non-endemic areas, and further expand the scope of the distribution of reservoir hosts. Few studies have focused on the infection of *S. japonicum* in wild animals. Future research could investigate the infection status among wild animals and explore the impacts of flooding on them to fill this gap and provide evidence for developing emergency responses to control schistosomiasis after flooding, although the investigation is fairly tough to carry out. Besides, it is hard to identify the time frame for zoonotic transmission during flooding. More specifically, previously infected animals, especially wild animals which are difficult to manage, could excrete feces into the flood water and the water will become contaminated. Furthermore, the fecal contaminated water will carry the parasite to infect snails and shed cercariae to infect healthy animals which contact the water during flooding. Thus, in response to this reciprocal causation of animal infection and fecal contamination, interventions should focus both on the management of animals and the treatment of the water.

### 2.3. The Impact of Flooding on Environmental Health Specifically for Oncomelania hupensis

Flooding will change the ecological environment. Water conservancy (dams, river embankments, channels, levees, etc.) and sanitary facilities can be damaged by severe floods. The damage of sanitary facilities may lead to a higher risk of fecal contamination. The main impact of flooding on the environment is the change in the density and distribution of *O. hupensis* since the snail is the crucial link in the life cycle of *S. japonicum* [51]. Without the existence of *O. hupensis*, the transmission of schistosomiasis will be interrupted successfully. This is the reason why snail control is crucial for schistosomiasis elimination [52].

The density of *O. hupensis* could be influenced by flooding, through the impact on development, reproduction, and ability to survive under the condition of submergence [53]. On the short-term individual level, continuous rainfall and the subsequent flooding facilitate the establishment of snail colonies on vegetation [47]. During rainfall and flooding, the egg production of the snails significantly increases, with one female producing on average two eggs in five days [54]. Yang et al. reviewed the data between 1995–2002 in Hunan province of China, and claimed that the annual rainfall, days of daily rainfall greater than 0.1 mm, and the days inundated with water (favorable for 2 to 7 months) were significantly associated with the reproduction of *O. hupensis* [55]. On the long-term population level, there is a trend of the pattern of snail density in certain local snail habitats [56]. Several studies reported that the rate of the frames with living snails fell in the first two years after flooding and then rose quickly from the third year [38,57]. The decrease in the first two years can be explained as due to the considerable deaths of adult snails [58] and the limited capacity of developing and hatching for snail eggs [59] under the condition of submergence. However, some adult snails can survive through a period of natural drowning and young snails developed well under the drowning condition [58]. This may be the reason for the subsequent increasing trend from the third year after flooding. A study reviewed the annual snail survey in Jiangsu province in China from 1998 to 2003 and reported similar trends in the rate of the frames with infected snails, the snail infection rate, and the density of infected snails [57]. This provides an explanation for the higher risk of schistosomiasis transmission with a higher density of total living snails.

The influence is also demonstrated in the active and passive diffusion of the snails for the following expanded snail habitats. When flooding occurs, the snails drown, climb trees, and passively float down rivers [60]. Along with the side-weir flow after the flood discharge, the snails stay in the places where flow velocity is small, such as the vortex areas [61]. New decent potential habitats develop with the mud deposition due to the flood flow and the snails can actively and slowly move to neighboring new habitats. These active and passive dispersals can result in the enlargement of previous snail habitats, the emergence of new snail habitats, and the rebound in previously snail eliminated areas [52]. During the period from 1979 to 2000, the re-emerging and newly discovered snail habitats in the flooding years accounted for up to 5.8% and 10.1% of the total snail habitats in the Yangtze River valley and were 2.6 and 2.7 times larger than the areas in years with normal hydrologic conditions, respectively [38]. After the 2016 catastrophic flood in China, the re-emerging and the newly discovered snail habitat areas in Anhui province were 1375 hm^2^ and 1288 hm^2^, respectively [62]. It should be noted that snail dispersal often presents as a retardation effect of flooding, since snails in the new habitats may not reproduce to a large amount that could be easily found by snail surveys and surveillance in one or two years after flooding.

## 3. The Strategies to Control and Prevent the Impacts of Flooding on the Transmission of Schistosomiasis from the One Health Perspective

Flooding will increase the risk of *S. japonicum* infection. For human and animals, behavior changes increase the intensity of water contact. For the environment, the occurrence of flooding always occurs along with the change of the previous aquatic environment and the climate. These changes will be reflected in the breeding, development, and distribution of the intermediate host snails, and then have an influence on the prevalence of schistosomiasis. Meanwhile, flooding will damage the dams and reservoirs in non-endemic areas which should have protected the residents from infection, and further cause the spread of snails, leading to larger snail habitats and higher risk of transmission probability. Therefore, specific intervention should be developed and implemented comprehensively to prevent the outbreak of schistosomiasis when flooding occurs.

### 3.1. For the Human Beings

School-based and community-based health education projects using various education models should be continuously implemented in the endemic areas of schistosomiasis [63]. People should be educated about the modes of transmission and risk behaviors [64,65]. Through education, residents are more likely to avoid unnecessary water contact as well as apply self-protection when they have to make contact with the flood water. Towards the major pattern of water contact, specific health education strategies should be applied to each age or sex group. Evidence has proven the effectiveness of interpersonal communication approaches to improve knowledge about schistosomiasis prevention and correct previous high-risk behaviors [66,67]. Effective health education should continue to the period even after flooding.

During flooding, unsafe water contact should be minimized. Safe and clean water are necessary for victims to make them stay away from the contaminated water. Proper feces management should be implemented to avoid water contamination. For people who participate in flood rescue and necessary production activities, adequate self-protection measures should be equipped before inevitable water contact. The measures include smearing anti-miracidia drugs and wearing rubber clothes, gloves, and shoes, which will keep people safe from the cercariae in the water. Moreover, all individuals potentially exposed to the contaminated water should be registered and followed up with early screening, early reporting, and early treatment.

After flooding, chemotherapy should be available to the affected population. Praziquantel (PZQ) is the only choice of chemotherapy for schistosomiasis, recommended by the World Health Organization and has been widely used for over 40 years. People who have water contact in high-risk areas during flooding should take early preventive chemotherapy with PZQ within four to five weeks to prevent acute infection [68]. The anti-schistosome effect could remain over time [69]. For people who participate in flood recuses and repeatedly contact the water, the combinations of PZQ and artemisinin derivatives provide higher protection than PZQ monotherapy [68].

### 3.2. For Animals

For domestic animals, the interventions before, during, and after flooding are consistent. First, risk monitoring systems should be continuously operated, especially after flooding. With the analysis of long-term data of routine surveillance, the risk factors and emergent cases can be easily detected and managed followed by a quick response. Then the transmission will be limited at the initial stage. Second, the number of domestic animals that serve as major reservoir hosts should be reduced significantly in endemic areas. Excessive animals represent more potential infection sources and higher transmission risk. Third, contact with unknown open water and potential endemic water should be reduced, and better, be avoided. Especially, the pasturing of livestock in marshland infested with *Oncomelania* should be prohibited, although grass production in the plains is greatly supported by flooding. Instead, herding in snail-free areas can be allowed. Fourth, feces hazard-free treatment should be issued to reduce the risk of water pollution caused by the excreted feces with eggs, to cut off the transmission chain. Finally, the same as human beings, preventive chemotherapy towards domestic animals after flooding should be especially carried out to reduce the disease burden of livestock.

For wild animals, currently there are no proper approaches for control and management [70]. This remains one of the challenges for transmission control during flooding and future goals of schistosomiasis elimination. Conducting surveillance on wild animals continuously, especially after flooding, is crucial to explore the hotspot and transmission tendency of schistosomiasis in the environment, thus providing references for policymaking. In China, the current strategy towards schistosomiasis control in wild animals is to indirectly monitor the infection status of wild feces by the ministry of health. From the One Health perspective, cross-sectoral cooperation between the ministry of health, agriculture, and forestry is needed to routinely capture sentinel mice or other wild animals for monitoring of the infection situation.

### 3.3. For the Environment

Before flooding, better levee, enlarged reservoirs, improved early warning systems, and accurate hazard risk evaluations are needed to control the severe health and economic losses of flooding [71]. The earliest warning of flooding can be offered via communication and cooperation with the ministry of the meteorological administration, which will offer sufficient time for disease control departments to respond to the coming flooding in preparation for dealing with schistosomiasis transmission. The implementation of integrated water resource management is a sustainable approach to protect local residents and animals from the effects of flooding and prevent the breeding and dispersal of snails [72]. For instance, creation and improvement of dam reservoirs can decrease the risk of embankment collapse and ameliorate water supply and sanitation. The Three Gorges Dam significantly reduced the frequency of high-water levels that were beyond the warning level [73]. The dam reservoirs together with the irrigation systems can break the transmission cycle of schistosomiasis by reducing human-water contact, diminishing environmental contamination with excreta, and decreasing the density of the living snails [73,74]. However, it should be noted that the construction of the water conservancy project may also expand the snail habitats and result in the emergence of new transmission sites without appropriate anti-schistosomiasis measures. A meta-analysis reviewed studies about the association between the prevalence of schistosomiasis and the dam construction in Africa and reported a pooled risk ratio of 2.4 (95%CI 1.4–3.9) and 2.6 (95%CI 1.4–5.0) for the infection of *S. haematobium* and *S. mansoni*, respectively [74]. To date, these has been no similar report regarding *S. japonicum* prevalence. Thus, when considering a strategy from the One Health perspective, the water conservancy program for schistosomiasis control should combine with snail control approaches [75] and other interventions towards human and animals, such as health education and the control for all species of infection sources, to ensure effective measures for a comprehensive strategy. This intervention calls for cooperation between the ministry of health and water resources.

During flooding, temporary hygiene and sanitary facilities should be built in time. The toilets in crowd gathering areas where displaced people live should be constructed in a timely manner. The supply of safe and clean water could effectively prevent water-borne diseases, including schistosomiasis. Besides, human and livestock feces should be put through hazard-free treatment to avoid contamination. In addition, warning signs should be displayed in the most visible places to warn people to stay away from the risky areas.

After flooding, the damage of banks, water supply and sanitation facilities should be assessed. Priorities should be given to repairing damaged embankments and other water infrastructures. Then, drugs for killing cercaria should be discharged into the waterbody to preventively control the existence of schistosomes. Most importantly, snail control should be carried out to interrupt the transmission after flooding [76]. Repeated use of molluscicide is an effective strategy to reduce the density of *Oncomelania* and plays a key role in the elimination of schistosomiasis. Although niclosamide is reported to be toxic to fish and damaging to the environment and biodiversity, it is still the only molluscicide recommended by WHO, which is highly active against all stages of the snail life cycle, as well as schistosome larvae [77]. To advance precise snail control, remote sensing and geographic information system (GIS) technologies could be utilized as potentially powerful methods to simulate flood inundation, map flood dynamics, predict water levels, and forecast the spatial distribution of snails and the risk areas of schistosomiasis before and post flooding [78,79,80,81,82]. Using the data from Sentinel-1A and 1B, and Landsat 8 SAR remote sensing images, the accuracy of the model to predict the snail distribution during and after flooding is estimated to be around 85% [83,84]. By accurately predicting the snail distribution, the model could be a strong approach to timely identify the areas with higher transmission risk of schistosomiasis and provide a hint for targeted responses after flooding.

The strategies for the environment before, during, and after flooding may be applied in Africa for *S. mansoni* and *S. haematobium* control since all human- hosting *Schistosoma* spp. have a similar life cycle, although their intermediate hosts and major definitive hosts are diverse. The early warning of flooding, setting up warning signs and killing cercaria in risky areas during or post flooding, reconstructing water conservancy, developing hygiene and sanitary facilities, conducting snail surveillance, using molluscicide, and predicting the risky transmission areas after flooding can also be effective.

## 4. Conclusions and Next Steps

In this article, we reviewed the impact of flooding on the transmission of schistosomiasis japonica in human beings, animals, and the environment. Flooding damages water conservancy and sanitary facilities and increases the intensity of water contact for both humans and animals, which further increases the infection risk of *S. japonicum*. In addition to the impact of inundated conditions on the development and reproduction of *Oncomelania*, flooding may carry snails to previously snail-free areas and lead to the active and passive diffusion of the snails. As a result, the density of snails will significantly increase from the third year after flooding [38,57], and snail habitats can be enlarged due to the diffusion. With more water contact from human and other reservoir hosts, and larger snail habitats with higher density of living snails, the transmission risk of schistosomiasis increases under the influence of flooding, especially catastrophic flooding.

With the agenda set for the global schistosomiasis elimination by 2030, considering the life cycle of schistosomes and transmission features of schistosomiasis japonica, strategies for intervention must be developed and implemented with the context of One Health, responding to the impacts of flooding effectively. For humans, health education, adequate self-protection equipment, preventive chemotherapy, safe water supplements, and early case screening, reporting, and treatment will protect people from infection and prevent the rebound of schistosomiasis. For animals, culling susceptible domestic animals, herding in snail-free areas, proper feces treatment, and continuous surveillance, including of wild animals, should be carried out to block the transmission chain. For the environment, early warnings of flooding, setting up warning signs and killing cercaria in risky areas during or post flooding, reconstructing water conservancy, developing hygiene and sanitary facilities, conducting snail surveillance, using molluscicide, and predicting the risky transmission areas after flooding all contribute to reducing schistosomiasis transmission risk. The implementation of these strategies needs multi-sectoral cooperation to achieve the goal of minimizing the adverse impacts of flooding. In addition, there are still topics in need of discussion, such as wild animal management and the long-term impact of flooding on the spread of snails. Further research could provide more evidence for better control of flooding impacts on schistosomiasis.
